# Methods and evidence in economic evaluations of modern radiotherapy: a systematic mapping review protocol

**DOI:** 10.1136/bmjopen-2026-119043

**Published:** 2026-09-22

**Authors:** Fabio Veneruso, Anna Barnes, Christian Graeff, Heidi Probst, Michael Schlander, Tracy Underwood, Lennart Volz, Vincent Grégoire, Lionel Perrier

**Affiliations:** 1 Centre Leon Berard, Lyon, France; 2 GATE-LSE, Lyon, France; 3 King’s Technology Evaluation Centre, King’s College London, London, UK; 4 Biophysics Department, GSI Helmholtzzentrum für Schwerionenforschung GmbH, Darmstadt, Germany; 5 Sheffield Hallam, Sheffield Hallam University College of Health Wellbeing and Life Sciences, Sheffield, UK; 6 Institute for Innovation and Valuation in Health Care, Wiesbaden, Germany; 7 Department of Medical Physics & Biomedical Engineering, University College London, London, UK; 8 GSI Helmholtzzentrum für Schwerionenforschung GmbH, Darmstadt, Germany

**Keywords:** Radiation oncology, HEALTH ECONOMICS, Cancer, Health Care Costs

## Abstract

**Abstract:**

**Introduction:**

Modern radiotherapy modalities have increased treatment precision and clinical effectiveness. However, the economic evidence that underpins their adoption is highly heterogeneous, with substantial variation in costing methods, data sources, analytic designs (trial-based, real-world/observational and decision-analytic modelling), perspectives and the treatment of uncertainty. We propose a systematic mapping review to characterise the peer-reviewed evidence base and identify methodological gaps relevant to health technology assessment and policy-making. This systematic mapping review aims to: (1) systematically catalogue the published, peer-reviewed evidence and the methodological approaches used in economic evaluations of modern radiotherapy; (2) identify methodological trends, including the uptake and implementation of value of information analysis and (3) highlight evidence gaps by tumour type, geographical region and methodological approach.

**Methods and analysis:**

Following the Preferred Reporting Items for Systematic reviews and Meta-Analyses extension for Scoping Reviews (PRISMA-ScR) guidelines, we searched PubMed (MEDLINE), Embase, Scopus, Web of Science Core Collection and EconLit for economic evaluations of radiotherapy published from 2005 onwards, with an initial search on 24 February 2026 and a planned search update before synthesis. Study selection is ongoing; extraction and quality appraisal in duplicate have not been undertaken and no results are reported. Reporting completeness, methodological limitations and uncertainty will be appraised as three separate dimensions using the Consolidated Health Economic Evaluation Reporting Standards (CHEERS) 2022 checklist, the Bias in Economic Evaluation (ECOBIAS) checklist and the TRansparent Uncertainty ASsessmenT (TRUST) framework, applied descriptively and not combined into a single quality score. Methodological information will be extracted, including model structure (eg, Markov model), analytical perspective (eg, healthcare payer), time horizon, costing approach (eg, diagnosis-related group (DRG)-based) and utility measurement (eg, EQ-5D). The methods used to assess uncertainty will be mapped for all studies, with the ECOBIAS checklist and the TRUST framework only applied to decision-analytic model-based evaluations. Findings will be synthesised descriptively as an evidence gap map and visualised as heat maps and bubble plots. A conceptual co-occurrence network, analysed in R with visualisation in VOSviewer, will map how modalities, tumour sites and economic characteristics cluster across studies. No meta-analysis is planned, given the expected heterogeneity of our findings.

**Ethics and dissemination:**

This review does not require ethical approval, as it will use data from previously published studies. The results will be disseminated through peer-reviewed publications, conference presentations and open-access releases of data extraction files and visual maps in the Open Science Framework (OSF).

**Registration:**

This review protocol was prospectively registered (9 February 2026) in the OSF Registries (DOI: 10.17605/OSF.IO/6W2TJ).

**STRENGTHS AND LIMITATIONS OF THIS STUDY**

- This review draws on a comprehensive search of five bibliographic databases, with reproducible deduplication and duplicate independent screening, full-text selection and data extraction.
- The review will apply a structured, pre-specified extraction framework characterising each study’s type of economic evaluation, model structure, analytical perspective, time horizon, discounting, costing approach (including micro-costing and time-driven activity-based costing), utility measurement and handling of uncertainty.
- Reporting completeness, methodological limitations and uncertainty will be appraised as three separate dimensions using the Consolidated Health Economic Evaluation Reporting Standards (CHEERS) 2022 checklist, the ECOBIAS checklist and the TRansparent Uncertainty ASsessmenT framework, with the latter two applied to decision-analytic model-based evaluations only and never combined into a composite score.
- As a descriptive mapping review, it will not include quantitative synthesis or meta-analysis, thus precluding pooled estimates or ranking of the cost-effectiveness of modalities.
- Eligibility will be restricted to peer-reviewed, English-language publications, excluding grey literature, which may under-represent economic methods used in some real-world health technology assessment contexts.

## Introduction

 Radiotherapy is a central component of modern cancer management and contributes substantially to both curative treatment and symptom control across a wide range of tumour types.^1–3^ Over the past 20 years, an array of new and highly consistent delivery techniques has been developed, including intensity-modulated radiotherapy (IMRT), volumetric-modulated arc therapy (VMAT), stereotactic body radiotherapy (SBRT), stereotactic radiosurgery (SRS), proton therapy and carbon ion therapy.^4–7^ These techniques can allow for dose escalation, better tumour coverage and/or improved sparing of normal tissues in clinical practice, thus widening the therapeutic window.^8–10^ Their use comes with significant financial and organisational costs related to infrastructure (eg, shielded treatment rooms specifically designed for large equipment), purchasing and servicing the equipment needed, investment in planning and quality-assurance systems, increased planning complexity, workforce training and often reconfiguration of clinical workflows.^11 12^ Planning processes for IMRT and intensity-modulated proton therapy are substantially more complex and resource-intensive, although emerging automation tools, including artificial intelligence-based planning, may partially offset these requirements.^13–15^ Another important and complex aspect is fractionation, where hypofractionated regimens require greater precision at each step but shorten the overall treatment course. Thus, decisions regarding the implementation and scaling of these modalities have significant implications for health system resource allocation and long-term strategic planning.^16 17^

As a consequence, a number of economic evaluations have been performed to assess the value for money of modern radiotherapy techniques.^18^ Despite this growing body of research, the current evidence is typified by significant methodological heterogeneity,^19^ encompassing differences in study design, analytical perspective, time horizons, discounting practices and approaches to cost identification and measurement. Costing strategies are highly heterogeneous, ranging from gross or diagnosis-related group (DRG) costing to micro-costing and time-driven activity-based costing (TDABC), all of which capture resource use with varying degrees of accuracy and context-specificity.^19–22^ Utility measurement methods also vary, with studies drawing on generic preference-based instruments, including EQ-5D, condition-specific measures or expert-derived utility estimates that complicate comparability across evaluations.^18 23–25^

A critical dimension of methodological variation is the handling of uncertainty. Many studies perform deterministic sensitivity analyses; few, however, undertake probabilistic sensitivity analyses and only a minority incorporate value of information (VOI) methods, which can uniquely inform the value of further research.^26 27^ Moreover, beyond the level of analysis performed, sources of uncertainty, whether parameter, structural or methodological, are seldom acknowledged, let alone explicitly described.^28^ The TRansparent Uncertainty ASsessmenT (TRUST) framework provides a structured approach for identifying and classifying these sources, ensuring transparency and interpretability.^29^

Previous reviews in this area have typically focused on single radiotherapy modalities^24 25^ and specific tumour sites^30–32^ or have narrowly defined methodological questions, including cost approaches, stand-alone cost analyses and the quality of reporting,^19 20 33^ and therefore provide only a fragmented and selective picture of the underlying evidence base. A preliminary search of the Open Science Framework (OSF) registries (date: 10 December 2025) did not identify any ongoing systematic reviews or evidence-mapping reviews with the same scope and objectives. To our knowledge, no review has systematically described how economic evaluations in radiotherapy are designed across technologies, diseases and health system contexts or how different forms of uncertainty are conceptualised and addressed. We therefore propose a systematic mapping review to characterise the breadth of the published peer-reviewed evidence, identify methodological patterns and highlight persisting evidence gaps and decision-relevant uncertainties. Mapping reviews aim to identify, classify and visually display the distribution and characteristics of existing evidence rather than aggregate quantitative findings^34–37^ and are therefore particularly appropriate when the literature is conceptually diverse and methodologically heterogeneous.^38–40^ This review will provide a systematic overview of economic evaluations of modern radiotherapy by: (i) mapping the clinical focus and methodological characteristics of the included studies; (ii) classifying how uncertainty is handled, including the application of the TRUST framework and (iii) developing maps of evidence gaps and conceptual co-occurrence to describe research clusters, methodological trends and areas where further primary research or methodological standardisation is needed to support robust, decision-relevant economic evaluation in radiotherapy.

### Eligibility criteria and research questions

Eligible study designs will be classified following an established taxonomy of economic evaluation study designs^41 42^: (i) full economic evaluations, which compare both the costs and the consequences of two or more alternatives, namely cost-effectiveness, cost-utility and cost-benefit analyses, cost-minimisation analyses (only where equivalence of outcomes is explicitly demonstrated) and cost-consequence analyses (which report costs alongside multiple disaggregated outcomes); (ii) partial economic evaluations that do not jointly compare costs and consequences against a comparator, namely comparative cost analyses and single-arm costing studies and (iii) budget impact analyses, classified separately as assessments of affordability rather than efficiency. Single-arm costing studies will be eligible only if they explicitly quantify resource use or cost structures attributable to a defined modern radiotherapy modality. Studies must assess at least one modern radiotherapy modality as the decision-relevant technology under economic evaluation. Modern radiotherapy modalities are defined as advanced external beam delivery techniques, including IMRT, VMAT, SBRT/SRS, proton therapy, carbon ion therapy and magnetic resonance- or CT-guided adaptive radiotherapy. Studies in which three-dimensional conformal radiation therapy (3D-CRT) or brachytherapy are used as comparators to these modern modalities will be eligible, whereas studies evaluating 3D-CRT or brachytherapy alone will be excluded. Studies in which modern radiotherapy is delivered in combination with systemic therapy (eg, chemotherapy, androgen deprivation therapy or targeted agents) will be eligible, provided that the radiotherapy modality itself represents the primary technological comparator driving the economic analysis. Conversely, studies comparing broad multimodal therapeutic strategies (eg, surgery vs chemoradiation, active surveillance vs radiotherapy) will be excluded unless the radiotherapy modality is explicitly specified and constitutes the core technological element under evaluation. Eligible studies may be trial-based, model-based or real-world/observational economic evaluations and may adopt any analytical perspective: hospital, payer or societal. To ensure relevance to current practice, we will include studies published from 2005 to the date of the final search (24 February 2026 with a planned search update before synthesis) written in English and reporting original quantitative economic evaluations. The year 2005 marks the end of the transition from conventional radiotherapy to 3D-CRT and the beginning of the widespread clinical implementation of intensity-modulated and image-guided techniques, often referred to as ‘modern radiotherapy’.^7 8 43–45^

We will exclude editorials, letters, comments, narrative overviews, systematic reviews and meta-analyses (which will be used only for citation tracking), conference abstracts without full methodology, purely clinical studies without cost, treatment-planning comparisons without economic components and dosimetric-only studies. This review will seek to answer the following research questions.

#### Which areas of modern radiotherapy have been subject to economic evaluation?

This question aims to identify how the existing economic evidence is distributed across tumour sites, radiotherapy modalities (including IMRT, VMAT, SBRT/SRS, proton therapy, carbon ion therapy and MR- or CT-guided adaptive radiotherapy), clinical indications and geographical settings. Our objective is to capture patterns in the focus of economic research and highlight domains in which evidence remains limited or absent.

#### How are economic evaluations of modern radiotherapy conducted?

This question examines the methodological approaches used in economic evaluations, including the type of economic evaluation (classified as a full economic evaluation, partial economic evaluation or budget impact analysis), the underlying model structure (such as trial-based analysis, decision tree, Markov model or microsimulation), the analytical perspective, the time horizon and discounting strategy, the costing approach employed (eg, micro-costing, DRG/gross costing or TDABC), the methods used to derive health state utilities and the extent of uncertainty analysis, classified as absent, deterministic, probabilistic or incorporating the VOI methods. Table 1 reports inclusion and exclusion criteria.

**Table 1:** Inclusion and exclusion criteria for study selection

Component	Description
Population	Studies involving patients with cancer who are in receipt of modern external beam radiotherapy or model-based hypothetical cohorts representing such populations, where the radiotherapy modality constitutes the decision-relevant technology under economic evaluation.
Concept	Studies were classified, following an established taxonomy of economic evaluation study designs, as: (i) full economic evaluations (cost-effectiveness, cost-utility, cost-benefit, cost-minimisation with demonstrated outcome equivalence and cost-consequence analyses); (ii) partial economic evaluations (comparative cost analyses and single-arm costing studies) or (iii) budget impact analyses, classified separately as assessments of affordability. A direct link between the evaluated radiotherapy modality and the cost structure is required.
Context	Economic evaluations of modern external beam radiotherapy delivery modalities, including IMRT, VMAT, SBRT/SRS, proton therapy, carbon ion therapy and MR- or CT-guided adaptive radiotherapy. Image guidance (IGRT), respiratory gating, MRI-only workflows or other technical components will be considered eligible only when evaluated as integral elements of an eligible delivery modality and when they represent the technological focus of the economic analysis. Any health system setting and any analytical perspective.

The inclusion of IGRT terms aims to improve recall at the search stage. Eligibility and classification will be based on the underlying radiotherapy modality rather than on IGRT alone.

adaptive radiotherapy=modification of the treatment plan during the treatment course on the basis of on-board MR or CT imaging.

IGRT, image-guided radiotherapy; IMRT, intensity-modulated radiotherapy; PCC, population, concept and context; SBRT, stereotactic body radiotherapy; SRS, stereotactic radiosurgery; VMAT, volumetric modulated arc therapy.

## Methods and analysis

This study is designed as a systematic mapping review within the broader family of scoping reviews, consistent with the typology proposed by Grant and Booth.^37^ The objective is to describe, classify and visually characterise the distribution and methodological features of the existing evidence base rather than to synthesise effect estimates or perform quantitative pooling. This protocol is reported in accordance with the Preferred Reporting Items for Systematic Review and Meta-Analysis Protocols (PRISMA-P) checklist presented in online supplemental file 1.^46^ The review will be conducted in accordance with methodological guidance from the Joanna Briggs Institute^47 48^ and reported following the PRISMA-ScR checklist.^40^ It is further informed by recent methodological frameworks on mapping reviews, evidence maps and gap maps.^49^

### Databases and search strategy

A comprehensive literature search was conducted to identify economic evaluations of modern radiotherapy. The following electronic databases were searched from 1 January 2005 to 24 February 2026: PubMed (MEDLINE), Embase (Elsevier), Scopus, Web of Science Core Collection and EconLit (EBSCO). This review is deliberately restricted to the peer-reviewed evidence base; grey literature and health technology assessment (HTA) reports were not searched. This restriction provides a clearly defined corpus but may omit economic methods used in commissioned or jurisdiction-specific decision-making contexts. The search strategy combined free-text terms searched within the primary bibliographic fields of each database (eg, title, abstract and author keywords) with database-specific indexing terms, covering two main conceptual categories: (1) economic evaluations and cost and (2) radiotherapy modalities. Where supported by the database, results were limited to document type ‘Article’ to exclude conference materials and non-peer-reviewed records (eg, in Scopus, Web of Science and Embase). No restrictions were applied with respect to tumour type or disease stage at the search stage, and classification by tumour site occurred during data extraction.

To maximise sensitivity at the retrieval stage, the search strategy deliberately included broader radiotherapy-related terms (eg, hypofractionation, conformal radiotherapy, 3D-CRT, LINAC, radiosurgery). Eligibility and classification were subsequently restricted at the screening stage to modern external beam radiotherapy modalities as defined in the inclusion criteria. The presence of broader technical terms at the search stage does not imply automatic eligibility but serves to reduce the risk of missing relevant economic evaluations in which modern modalities are embedded within wider radiotherapy terminology.

An example search strategy for PubMed is provided in table 2.

**Table 2:** Example PubMed search strategy

Aspect	PubMed search strategy
Economic evaluation terms	((“cost-effectiveness”(Title/Abstract)OR “cost utility”(Title/Abstract)OR “cost-utility”(Title/Abstract)OR “cost benefit”(Title/Abstract)OR “cost-benefit”(Title/Abstract)OR “cost comparison”(Title/Abstract)OR “micro-cost*”(Title/Abstract)OR “economic evaluation”(Title/Abstract)OR “economic analys*”(Title/Abstract)OR “cost analys*”(Title/Abstract)OR “cost minimization”(Title/Abstract)OR “cost consequence”(Title/Abstract)OR “budget impact”(Title/Abstract)OR “hospital cost*”(Title/Abstract)OR “treatment cost*”(Title/Abstract)OR “healthcare cost*”(Title/Abstract)OR “TDABC”(Title/Abstract)OR “incremental cost-effectiveness ratio”(Title/Abstract)OR ICER(Title/Abstract)OR “probabilistic sensitivity analysis”(Title/Abstract)OR “value of information”(Title/Abstract)OR VOI(Title/Abstract)) OR (“Costs and Cost Analysis”(MeSH))OR “Cost-Benefit Analysis”(MeSH))OR “Cost-Effectiveness Analysis”(MeSH)))
Radiotherapy terms	((radiotherapy(Title/Abstract)OR “radiation therapy”(Title/Abstract)OR “radiation oncology”(Title/Abstract)OR “adaptive RT”(Title/Abstract)OR IMRT(Title/Abstract)OR “field in field”(Title/Abstract)OR VMAT(Title/Abstract)OR IGRT(Title/Abstract)OR “MR-linac”(Title/Abstract)OR SBRT(Title/Abstract)OR “stereotactic radiosurgery”(Title/Abstract)OR SRS(Title/Abstract)OR “proton therapy”(Title/Abstract)OR “particle therapy”(Title/Abstract)OR “hadron therapy”(Title/Abstract)OR “carbon ion”(Title/Abstract)OR “heavy ion”(Title/Abstract)OR EBRT(Title/Abstract)OR “external beam”(Title/Abstract)OR “conformal radiotherapy”(Title/Abstract)OR “3D-CRT”(Title/Abstract)OR “hypofractionation”(Title/Abstract)OR LINAC(Title/Abstract)OR “gamma knife”(Title/Abstract)OR “CyberKnife”(Title/Abstract)OR radiosurgery(Title/Abstract)) OR (“Radiotherapy”(MeSH))OR “Radiotherapy, Intensity-Modulated”(MeSH))OR “Radiotherapy, Image-Guided”(MeSH))OR “Proton Therapy”(MeSH))OR “Heavy Ion Radiotherapy”(MeSH)))
Final combined query	#1 AND #2

3D-CRT, three-dimensional conformal radiation therapy; EBRT, external beam radiotherapy; ICER, incremental cost-effectiveness ratios; IGRT, image-guided radiotherapy; IMRT, intensity-modulated radiotherapy; MeSH, Medical Subject Headings; RT, radiotherapy; SBRT, stereotactic body radiotherapy; SRS, stereotactic radiosurgery; TDABC, time-driven activity-based costing; VMAT, volumetric-modulated arc therapy; VOI, value of information.

Search strategies were tailored to each database, taking into account variations in indexing structures and vocabulary usage. Free-text searching across primary bibliographic fields was prioritised, due to known heterogeneity in indexing of radiotherapy modalities and economic evaluation terminology across databases. Controlled vocabulary (eg, MeSH and Emtree) was incorporated where available to enhance sensitivity. In addition to electronic database searching, backward and forward citation tracking of included studies and relevant reviews will be undertaken using an iterative pearl-growing approach until no further eligible studies are identified.^50^ Moreover, where recurring author names or key specialty journals are identified, targeted author- and journal-based searches will also be conducted to minimise the risk of missing relevant evidence. The complete search strategies for all five databases, including the exact strings, platforms, dates and numbers of records retrieved, are provided in online supplemental file 2). All database searches will be updated before synthesis, using the same strategies and the same interface limiters, restricted to records indexed after 24 February 2026 and deduplicated against the existing set. The date of the updated search, the number of additional records retrieved after deduplication and the number of additional studies included will be reported in the PRISMA flow diagram and in the completed review. The review is expected to be completed by 30 October 2026.

### Study selection

The results from all database searches were exported to Zotero for reference management and further processed using the automated systematic search deduplicator for duplicate removal in a transparent and reproducible manner.^51^ After deduplication, the dataset was imported into Rayyan^52^ for screening purposes. At registration (9 February 2026), no searches had been run yet, and no records had been screened. The search was executed on 24 February 2026, and title and abstract screening began on 25 February 2026, so the protocol was registered before study selection commenced. At the original submission on 2 March 2026, around 7% of the 6883 records remaining after deduplication had been screened. As of 31 August 2026, the first reviewer has screened all 6883 records, while the second reviewer is still working on the screening. The only amendment affecting eligibility was the extension of adaptive radiotherapy to CT-guided delivery, and all records screened before 10 July 2026 were re-examined against the amended definition. The amended taxonomy governs classification at extraction, and it will be applied to all included studies. A calibration exercise on a sample of records was conducted to ensure consistent application of the eligibility criteria. Inter-rater agreement during screening will be assessed and reported using Cohen’s kappa for the pilot phase.

Study selection will be reported according to PRISMA-ScR guidelines, using a PRISMA-style flow diagram adapted for scoping reviews.^40^ First, two reviewers will independently screen all retrieved record titles and abstracts against the predefined eligibility criteria. Articles deemed potentially relevant or of uncertain relevance will then undergo full-text screening by the same two reviewers. Any disagreements that might occur at either stage will be resolved through discussion and, if necessary, consultation with a third reviewer. Reasons for exclusion at the full-text stage will be recorded. The selection process will be summarised in a PRISMA flow diagram in accordance with PRISMA-ScR, detailing the number of records identified, screened, excluded and included in the final review.

### Patient and public involvement

Patients and the public were not involved in the design, conduct, reporting or dissemination of this protocol.

### Data items

A standardised data extraction form will be pilot-tested on a subset of the included studies to ensure consistency and clarity. Data will be independently extracted from each eligible study by two reviewers. Any disagreement between the reviewers will be resolved through discussion or consultation with a third reviewer. The extracted data will be organised to reflect four overarching descriptive domains: study characteristics, clinical context, radiotherapy modality and the structure and results of the economic evaluation.

For each study, publication details (author, year and country, which will be further categorised by geographical region and World Bank income group) and clinical information (tumour site, disease stage, concomitant chemotherapy, patient population, sample size or modelled cohort size, patient characteristics including age, sex, ethnicity where reported and care setting) will be recorded. Radiotherapy characteristics will include the modality under evaluation and the comparator, along with planning or fractionation details, where available. In studies evaluating multiple radiotherapy modalities or mixed interventions, data will be extracted and classified according to the specific modality evaluated, where separable. In all other cases, the study will be categorised as mixed-modality.

The economic analytic dimension will include the type of evaluation conducted, the analytic perspective adopted, the time horizon, discounting and the model structure used. Costing approaches will be documented by distinguishing bottom-up methods (eg, micro-costing, TDABC) from top-down approaches (eg, DRG-based gross costing, tariffs, reimbursement schedules or claims-based data). In addition, we will record whether individual cost components, unit costs and cost year are explicitly reported.^19–21^

Reported costs and cost-effectiveness outcomes, such as quality-adjusted life years, life years gained and incremental cost-effectiveness ratios, will be extracted in the original currency and cost year, without conversion or standardisation. Absolute cost values will therefore not be compared directly across jurisdictions or cost years. We will also record the type of evaluation according to the taxonomy defined in the eligibility criteria, distinguishing comparative cost analyses from single-arm costing studies among partial evaluations and whether each study applies an explicit willingness-to-pay or cost-effectiveness threshold and of what type (eg, a fixed jurisdictional value or a Gross Domestic Product (GDP) -based threshold), where reported. For each study, we will also record how health outcomes and utilities were derived by classifying the methods used (eg, direct elicitation techniques such as time trade-off or standard gamble, generic preference-based instruments such as EuroQol 5-dimension (EQ-5D-3L or EQ-5D-5L) or Short Form 6-Dimension, disease-specific utility measures or mapping from non-preference-based quality-of-life instruments), together with the population value set or tariff used and its jurisdiction, where reported. We will also record the source of effectiveness data (eg, randomised trial, observational study, meta-analysis or expert opinion), where reported. This synthesis does not aim to pool results statistically but rather to identify patterns, methodological tendencies and substantive evidence gaps to inform future research priorities and the development of economic evaluations in radiotherapy. A draft data extraction form is provided in online supplemental file 3); this will undergo pilot testing and refinement before full implementation.

### Quality appraisal

Although critical appraisal is not routinely undertaken in scoping and mapping reviews, we include it to characterise patterns in reporting completeness and risk of bias across economic evaluations and to support interpretation of methodological gaps (eg, reporting of costing approaches, cost components and uncertainty handling). Quality appraisal will not be used as an inclusion criterion and will be reported descriptively.

Reporting completeness, methodological limitations and the characterisation of uncertainty will be assessed as three separate dimensions and will not be combined into a single overall quality score. Reporting completeness will be assessed using the^53^ Consolidated Health Economic Evaluation Reporting Standards (CHEERS) 2022 checklist,^53^ applied to full economic evaluations, as CHEERS is designed for studies that compare both costs and outcomes. Risk of bias and methodological limitations will be evaluated using the ECOBIAS checklist, applied to decision-analytic model-based economic evaluations.^54^ Uncertainty will be assessed using the TRUST framework, applied to the same subset. For partial economic evaluations and budget impact analyses, CHEERS will not be computed and the number and proportion of studies in each design category will be reported descriptively. The denominator of each appraisal will be reported alongside its results. Within studies that are appraised, any individual item that does not apply will be coded as ‘not applicable’, with the proportion of such ratings reported descriptively. Two reviewers will independently assess the quality of each study. Any disagreements will be resolved through discussion and, if necessary, by consulting a third reviewer. Each study will be assessed item by item, and the results will be summarised using a traffic light visualisation system (green=fully met; yellow=partially met; red=not met), except for TRUST, which is dichotomous by design and records each source of uncertainty as present or absent within each model aspect. A summary table will present the distribution of ratings across studies, and a narrative synthesis will describe the common methodological strengths and limitations across the three dimensions. Particular attention will be given to the costing approach and reporting of cost components, appropriateness and justification of the model structure, extent and robustness of uncertainty analysis (including whether VOI methods had been undertaken) and completeness of reporting, as these aspects are central to the economic evaluation of radiotherapy technologies. No formal statistical assessment of publication bias across studies will be undertaken, given the mapping objective and the absence of quantitative synthesis. Likewise, no formal assessment of confidence in cumulative evidence (eg, Grading of Recommendations Assessment, Development and Evaluation - (GRADE)) will be performed.

### Synthesis and evidence mapping

Given the heterogeneity in the study design, clinical contexts and economic evaluation frameworks, meta-analysis is not feasible or conceptually justified. Instead, the synthesis will focus on mapping and characterising the structure, scope and methodological diversity of the evidence base. The unit of analysis is the individual publication. Where a publication reports multiple radiotherapy modalities or cost estimates, these will be recorded as descriptive sub-strata nested within the parent publication. Counts and proportions in the evidence maps will therefore be computed at the publication level, and modality-level breakdowns will be presented descriptively. A single publication may contribute to more than one category, so that category totals may exceed the number of included studies. The first stage of the synthesis will entail tabulation of key study characteristics and descriptive summaries of the distribution of radiotherapy modalities, tumour sites, economic evaluation types, model structure and costing approaches. These descriptive summaries will be supplemented with heatmaps, bubble plots and matrix-based visualisations that enable us to depict the density and distribution of evidence across clinical and methodological domains and to identify tumour-technology pairs or evaluation approaches for which economic evidence remains limited or absent.

To examine how radiotherapy modalities, tumour indications and choices in economic evaluation design tend to co-occur across the literature, we will conduct a conceptual co-occurrence network analysis. Each study will be represented as a set of conceptual attributes, including (i) the radiotherapy modality evaluated, (ii) the tumour site, (iii) the type of economic evaluation, (iv) the model structure, (v) the costing method employed and (vi) the approach used to handle uncertainty.

The TRUST framework will be applied to decision-analytic model-based economic evaluations only, to classify parametric, structural and methodological sources of uncertainty.^29^ For non-model-based studies (trial-based or observational), TRUST will be recorded as not applicable. However, the reported uncertainty analysis methods (eg, deterministic sensitivity analysis, probabilistic sensitivity analysis, scenario analysis, threshold analysis and VOI approaches) will be extracted and mapped descriptively across all study designs.^28^ This approach allows us to distinguish where uncertainty originates in model-based evaluations and how it is handled across the broader body of evidence.

The co-occurrence matrix will be constructed at the study level from these coded attributes, and the network will be built and analysed in R using the igraph package. Nodes represent coded concepts and edges indicate the co-occurrence of two concepts within the same publication. Two edge weights will be distinguished and reported separately: the raw co-occurrence frequency, that is, the number of publications in which two concepts appear together; and the association strength, in which the observed co-occurrence is divided by the product of the total occurrences of the two concepts, so that edges are not inflated by the marginal frequency of common concepts such as IMRT. The raw co-occurrence matrix will be deposited on OSF, while community detection and the network layouts will be computed on the association-strength weights; the weighting used will be stated in each figure legend. Edges therefore denote joint occurrence rather than economic comparisons, which preserves the publication-level unit of analysis. Only concepts occurring in at least five studies will be retained. Thematic clusters will be identified with a modularity-based community-detection algorithm (the Leiden algorithm^55^), with the random seed fixed in advance and the resolution parameter reported alongside the results. The pairings of primary interest are: radiotherapy modality by type of economic evaluation and tumour site by radiotherapy modality. The distribution of modalities over publication year will instead be presented as a heatmap, since calendar year is a temporal axis rather than a conceptual node. Network maps will be visualised in VOSviewer^56^ from the same matrix, using association-strength normalisation, which is the VOSviewer default, so that both representations are based on the same normalised edge weights. The matrix, the input files and the analysis code will be deposited on OSF.

## Discussion

Modern radiotherapy encompasses a broad range of techniques that vary significantly in their clinical indications, workflow complexity, resource requirements and long-term cost implications.^11 12 22^ For healthcare systems under increasing budgetary pressure and faced with an expanding demand for radiotherapy services, the economic evidence to support the adoption and scaling of these modalities has become strategically important in informing decision-making and resource allocation.^12 17 22 57 58^ However, there is substantial methodological heterogeneity in the current evidence. Analytical perspectives, time horizons, costing approaches, model structures and outcome valuations all vary, while the treatment of uncertainty ranges from no sensitivity analysis to full probabilistic and rarely VOI analysis. These inconsistencies limit comparability across evaluations and constrain the transferability of findings between institutions, jurisdictions and health system contexts. This systematic mapping review is designed to deliver a broad, structured overview of the methods and findings reported in the peer-reviewed economic evaluation literature on modern radiotherapy. Cataloguing the analytical choices of evaluation type, model structure, costing depth, utility measurement and approaches to uncertainty in the studies included in this review will reveal recurring methodological patterns and gaps in rigour and transparency. The TRUST framework will be applied to model-based economic evaluations to ascertain whether uncertainty is primarily parameter-based, structural or methodological, thus suggesting where decision-relevant uncertainty is left unaddressed and where future analyses may benefit from a deeper examination.

Conceptual co-occurrence network analysis will facilitate visualisation of how radiotherapy modalities, tumour sites and methodological choices cluster in the literature. This approach not only reveals areas where economic evidence is concentrated, that is, those with well-developed evaluative practice, but also indicates sparse or conceptually isolated parts of the evidence base. Such visual and structural mapping will support the formulation of targeted research priorities, for example, identifying where new cost-effectiveness evaluations or VOI analyses may yield the greatest incremental value for policy-makers and HTA bodies. The focus is on describing the breadth, structure and methodological characteristics of the evidence base. This approach differs from traditional bibliometric analyses, which primarily examine co-authorship and institutional collaboration networks. Instead, our focus is on mapping the underlying conceptual framework of economic research in radiotherapy and identifying how clinical, technological and methodological elements are structured, clustered and linked across published evaluations. By identifying both densely clustered areas of evidence and thinly connected or missing conceptual links, the synthesis will show where economic evidence is concentrated and where it is sparse or absent, and where further methodological work, primary research or standardisation is required. In doing so, evidence mapping will inform future research priorities, methodological harmonisation efforts and the design of more rigorous economic evaluations in radiotherapy. Characterising where the evidence base is robust enough to inform policy or reimbursement decisions, however, falls outside the scope of a descriptive mapping review.

The extent of clinical, contextual and methodological heterogeneity limits comparability across studies and supports the use of a mapping rather than a quantitative synthesis approach. Our aim is to clarify the structure, maturity and coherence of the economic evidence base in radiotherapy, thereby supporting greater methodological consistency in future economic evaluations.

This review will have some limitations. First, by restricting it to English-language publications, our findings may under-represent economic evidence from non-English-speaking settings. Second, the exclusion of HTA reports and other grey literature, consistent with the review’s focus on the peer-reviewed evidence base, may underestimate the use of advanced economic evaluation methods, such as VOI analyses, in real-world decision-making contexts. Finally, the high heterogeneity in tumour sites, comparators, costing approaches and model structures precludes quantitative synthesis, limits direct comparability across studies and prevents cross-jurisdictional comparisons of cost-effectiveness.

This work is intended to support policymakers, guideline developers and HTA organisations in interpreting current evidence and prioritising those areas where further empirical and methodological investment is warranted and where clearer guidance on cost-effective research directions may be most valuable.

## Ethics and dissemination

As this study will use previously published data and will not involve human participants or the collection of new patient-level information, ethical approval is not required. This review will be conducted and reported in accordance with the PRISMA-ScR guidelines. These findings will be disseminated through publication in a peer-reviewed journal and presented at relevant academic and clinical conferences. All extracted datasets, data extraction forms and search strategies will be made publicly available through the OSF to support transparency and reproducibility.

### Amendments

The protocol was registered on 9 February 2026 and has since been amended two times: on 11 February 2026, to add Web of Science Core Collection to the databases searched; and on 10 July 2026, following peer review, to align the economic evaluation taxonomy, remove currency standardisation, add PRISMA-P reporting, expand the extraction framework and revise the completion date. Both amendments are documented and dated on the OSF registry. Any further amendments will be documented and dated on OSF, with the nature and rationale of each change clearly reported, and all substantial amendments will also be described in the final published review.

## Supplementary material

10.1136/bmjopen-2026-119043 online supplemental file 1

10.1136/bmjopen-2026-119043 online supplemental file 2

10.1136/bmjopen-2026-119043 online supplemental file 3
