# Supplementary material for: Methods and evidence in economic evaluations of modern radiotherapy: a systematic mapping review protocol
Source: BMJ Open. 2026 Sep 22;16(9):e119043. doi: 10.1136/bmjopen-2026-119043 (PMC13599855; doi:10.1136/bmjopen-2026-119043)
Supplement: Supplementary file 1 [file bmjopen-2026-119043.reviewer_comments.pdf]

PEER REVIEW HISTORY

BMJ Open publishes all reviews undertaken for accepted manuscripts. Reviewers are asked to complete a checklist review form and are provided with free text boxes to elaborate on their assessment. These free text comments are reproduced below.

ARTICLE DETAILS

Title (Provisional)

Methods and Evidence in Economic Evaluations of Modern Radiotherapy: A Systematic Mapping Review Protocol

Authors

Veneruso, Fabio; Barnes, Anna; Graeff, Christian; Probst, H.; Schlander, Michael; Underwood, Tracy; Volz, Lennart; Gregoire, Vincent; Perrier, Lionel

---

VERSION 1 - REVIEW

**Reviewer**	**1**
**Name**	**Ribeiro, Luis**
**Affiliation**	**Universidade do Algarve Escola Superior de Saude, Radiology**
**Date**	**12-Jun-2026**
**COI**	

Dear Authors,

Thank you for the opportunity to review this manuscript. It addresses a timely and potentially important topic. Modern radiotherapy technologies have substantial implications for capital investment, workforce requirements, treatment capacity, and healthcare expenditure. A systematic map of the economic evaluation literature could therefore be useful to health economists, radiotherapy researchers, health care providers, and health technology assessment bodies.

The manuscript has several strengths. The research area is relevant, the mapping-review design is broadly appropriate, the authors propose duplicate screening and data extraction, the protocol has been registered, and the planned extraction framework covers several important economic and technical dimensions. The attention given to costing approaches, model structures, health-state utility measurement, and uncertainty analysis is particularly valuable.

Nevertheless, this version requires major methodological revision. The principal concerns relate to the classification of economic studies, the reproducibility of the search, the operationalization of the appraisal frameworks, and the definition of the unit-of-analysis procedures.

The manuscript uses inconsistent tenses. The abstract indicates that the authors "will perform" the search, whereas the Methods section states that searches were conducted up to 24 February 2026, and that title and abstract screening began on 25 February 2026. Please report: the OSF registration date;

the review stage reached at registration; the number or proportion of records already screened at submission; whether full-text screening data extraction or appraisal had commenced; any differences between the registered and submitted protocols.

All completed procedures should be described in the past tense, and genuinely prospective procedures in the future tense.

Second, the manuscript classifies budget impact analyses, along with cost-effectiveness, cost-utility, cost-benefit, and cost-minimization analyses, as full economic evaluations. This is conceptually problematic.

A full economic evaluation compares both costs and consequences between alternatives. Budget impact analyses assess financial affordability within a particular budget and period and should normally be classified separately. Cost-minimisation analysis should only be considered appropriate where equivalent outcomes have been adequately demonstrated. Please establish a consistent taxonomy covering: full comparative economic evaluations; budget impact analyses; cost-consequence analyses; single-arm costing studies; other partial economic evaluations. This taxonomy should be applied consistently in the eligibility criteria, research questions, extraction form, and planned synthesis.

Third, reconsider or more clearly justify the exclusion of grey literature.

Fourth, provide fully reproducible search methods. Only an example PubMed strategy is included.

Fifth, refine the definition of modern radiotherapy. The scope of the review is broadly understandable, but several boundaries remain unclear.

Sixth, define the unit of analysis and prevent double-counting. The protocol does not state whether the unit of analysis is the publication, study, model, economic comparison, or radiotherapy modality.

Seventh, expand and revise the extraction framework. The extraction form should include additional variables relevant to radiotherapy economics, including: type of partial economic evaluation; incremental costs and outcomes; decision threshold; net monetary or health benefit; capital investment and amortisation; equipment lifetime; maintenance and quality-assurance cost; workforce and planning costs;...; jurisdiction and value set used for utilities. The Appendix currently does not adequately represent eligible single-arm cost studies, mixed-modality studies, or evaluations with no active comparator.

Please also clarify whether costs will be converted to a common currency and price year. If standardisation is planned, describe the inflation indices, conversion approach and reference year. If not, avoid direct comparisons of absolute costs between jurisdictions and periods.

Eight, Separate reporting quality, methodological limitations, and uncertainty.

Nineth, provide a reproducible network-analysis plan. The proposed VOSviewer is insufficiently described.

Tenth, moderate the policy and reimbursement claims. The manuscript states that the review will determine where the evidence is robust enough to support policy and reimbursement decisions. The proposed descriptive methods cannot necessarily support the conclusion.

In conclusion, this is a relevant and potentially useful protocol, and the systematic mapping approach is broadly suitable for the research objectives. However, substantial methodological clarification is required to ensure that the review is reproducible and that its eventual conclusions remain proportionate to the evidence generated.

I recommend major revision, with particular emphasis on the classification of economic evaluations, complete reporting of the search strategies, definition of the unit of analysis, operationalisation of CHEERS/ECOBIAS/TRUST, and detailed prespecification of the evidence-mapping and network-analysis methods.

Kind regards,

VERSION 1 - AUTHOR RESPONSE

REVIEWER 1 (Dr Luís Ribeiro)

Comment 1

The manuscript uses inconsistent tenses. Please report: the OSF registration date; the review stage reached at registration; the number or proportion of records already screened at submission; whether full-text screening, data extraction or appraisal had commenced; any differences between the registered and submitted protocols.

We apologise for these inconsistencies. The registration date (9 February 2026) has been added to the Registration statement of the abstract, and the stage reached at each point is now reported in the Study Selection section (p. 13, line 255 to p. 14, line 260):

> *"At registration (9 February 2026) no searches had been run yet, and no records had been screened. The search was executed on 24 February 2026 and title and abstract screening began on 25 February 2026, so the protocol was registered before study selection commenced. At submission on 2 March 2026, around 7% of the 6,883 records that remained after deduplication had been screened. Full-text screening, data extraction, and quality appraisal had not yet begun, and no results had been generated."*

Differences between the registered and the submitted protocol are reported in the Amendments section (p. 22, lines 455–463):

> *"The protocol was registered on 9 February 2026 and has since been amended twice: on 11 February 2026, to add Web of Science Core Collection to the databases searched; and on 10 July 2026, following peer review, to align the economic evaluation taxonomy, remove currency standardization, add PRISMA-P reporting, expand the extraction framework, and revise the completion date. Both amendments are documented and dated on the OSF registry. Any further amendments will be documented and dated on OSF, with the nature and rationale of each change clearly reported, and all substantial amendments will also be described in the final published review."*

Comment 2

The manuscript classifies budget impact analyses, along with cost-effectiveness, cost-utility, cost-benefit, and cost-minimization analyses, as full economic evaluations. This is conceptually problematic. Please establish a consistent taxonomy and apply it consistently in the eligibility criteria, research questions, extraction form, and planned synthesis.

We agree entirely: a budget impact analysis assesses affordability rather than efficiency, and cost-minimisation is defensible only where equivalence of outcomes has been shown. In the Eligibility criteria and research questions section, the passage

> *"We will include full economic evaluations (cost-effectiveness, cost-utility, cost-benefit, cost-minimization, and budget impact analyses) as well as structured partial economic evaluations (e.g., cost analyses, cost-consequence analyses), provided that a direct and explicit link between the evaluated radiotherapy modality and the cost structure is established."*

has been replaced by (p. 7, lines 150–158):

> *"Eligible study designs will be classified following an established taxonomy of economic evaluation study designs [41,42]: (i) full economic evaluations, which compare both the costs and the consequences of two or more alternatives, namely cost-effectiveness, cost-utility, and cost-benefit analyses, cost-minimization analyses (only where equivalence of outcomes is explicitly demonstrated), and cost-consequence analyses (which report costs alongside multiple disaggregated outcomes); (ii) partial economic evaluations that do not jointly compare costs and consequences against a comparator, namely comparative cost analyses and single-arm costing studies; and (iii) budget impact analyses, classified separately as assessments of affordability rather than efficiency."*

The same three categories now govern the second research question, the Concept row of Table 1, the 'Type of evaluation' item of Appendix 1, and the Quality Appraisal and Synthesis sections, where the choice of appraisal tool and the counts reported follow the same classification.

Comment 3

Reconsider or more clearly justify the exclusion of grey literature.

We have kept the exclusion but made its rationale explicit rather than implicit. The following has been added to the Databases and Search Strategy section (p. 10, lines 216–219):

> *"This review deliberately maps the peer-reviewed evidence base: grey literature and health technology assessment (HTA) sources are not consistently peer-reviewed and cannot be reproduced, rendering them incompatible with the reproducibility objective of the map; they were, therefore, not included."*

Because this is a deliberate restriction, its consequence is acknowledged as a limitation both in the 'Strengths and limitations' box and in the Discussion, where we note that excluding HTA reports may under-represent the use of advanced methods, such as value of information analysis, in real-world decision-making contexts.

Comment 4

Provide fully reproducible search methods. Only an example PubMed strategy is included.

We agree. The verbatim strategies as run for all five databases are now given in Supplementary File 1, each with the platform, search date, fields, limits and records retrieved, together with the deduplication step from 12,951 retrieved records to the 6,883 that entered screening. Publication-type limits (document type = "Article") were applied through the interface in Embase, Scopus and Web of Science and are recorded in the 'Filters applied' column, since interface limiters of this kind are not reproduced within the exported string. The following sentence has been added to the Databases and Search Strategy section (p. 13, lines 247–248):

> *"The complete search strategies for all five databases, including the exact strings, platforms, dates, and numbers of records retrieved, are provided in Supplementary File 1."*

Comment 5

Refine the definition of modern radiotherapy. The scope of the review is broadly understandable, but several boundaries remain unclear.

Two boundaries were genuinely unclear and have been resolved. Adaptive radiotherapy had been restricted to MR-guided delivery, which would have excluded CT-guided adaptive workflows that raise the same economic questions; CT-guided adaptive radiotherapy has therefore been added. Brachytherapy was not mentioned at all; it is now placed explicitly outside the definition, as an internal rather than external beam technique, while remaining admissible as a comparator. The definition now reads (p. 7, lines 161–166):

> *"Modern radiotherapy modalities are defined as advanced external beam delivery techniques, including IMRT, VMAT, SBRT/SRS, proton therapy, carbon ion therapy, and Magnetic Resonance- or CT-guided adaptive radiotherapy. Studies in which Three-Dimensional Conformal Radiation Therapy (3D-CRT) or brachytherapy are used as comparators to these modern modalities will be eligible, whereas studies evaluating 3D-CRT or brachytherapy alone will be excluded."*

The Context row of Table 1 and the 'Modality evaluated' item of Appendix 1 have been aligned with this definition.

Comment 6

Define the unit of analysis and prevent double-counting. The protocol does not state whether the unit of analysis is the publication, study, model, economic comparison, or radiotherapy modality.

The unit of analysis and the procedure for avoiding double-counting are now specified in the Synthesis and Evidence Mapping section (p. 17, line 351 to p. 18, line 356):

> *"The unit of analysis is the individual publication. Where a publication reports multiple radiotherapy modalities or cost estimates, these will be recorded as descriptive sub-strata nested within the parent publication. Counts and proportions in the evidence maps will therefore be computed at the publication level, and modality-level breakdowns will be presented descriptively. A single publication may contribute to more than one category, so that category totals may exceed the number of included studies."*

The same convention governs the network analysis, where edges are defined as co-occurrence within a single publication rather than as economic comparisons.

Comment 7

Expand and revise the extraction framework, to include type of partial economic evaluation, incremental costs and outcomes, decision threshold, net monetary or health benefit, capital investment and amortisation, equipment lifetime, maintenance and quality-assurance cost, workforce and planning cost, jurisdiction and value set used for utilities. Please also clarify whether costs will be converted to a common currency and price year.

On currency, we have decided not to convert to a common currency and price year, and to say so explicitly in the Data Items section (p. 15, lines 295–298):

> *"Reported costs and cost-effectiveness outcomes, such as quality-adjusted life years (QALYs), life years gained (LYG), and incremental cost-effectiveness ratios (ICERs), will be extracted in the original currency and cost year, without conversion or standardization. Absolute cost values will therefore not be compared directly across jurisdictions or cost years."*

The object of the review is the distribution of methods, not the magnitude of costs; converting values across jurisdictions and price years would rest on a contestable chain of assumptions and imply a comparability the data do not support. This deviation from the registered protocol is recorded in the OSF amendment of 10 July 2026.

The type of partial evaluation and the decision threshold have been added to the same section (p. 15, lines 298–302):

> *"We will also record the type of evaluation according to the taxonomy defined in the eligibility criteria, distinguishing comparative cost analyses from single-arm costing studies among partial evaluations, and whether each study applies an explicit willingness-to-pay or cost-effectiveness threshold, and of what type (for example, a fixed jurisdictional value or a GDP-based threshold), where reported."*

On utilities, the extraction now records the population value set or tariff and its jurisdiction (p. 16, lines 305–308), since two studies may report EQ-5D utilities while applying value sets from different countries. Incremental costs and outcomes are already captured in part: Appendix 1 records the reported cost values, the outcomes reported, and whether an ICER is reported with its value, so the incremental comparison on which each evaluation rests is documented, even though its two components are not stored as separate numerical fields. Net monetary and net health benefit are not extracted as separate items.

On capital investment and amortisation, equipment lifetime, maintenance and quality-assurance costs, and workforce and planning costs, we have considered the suggestion but decided not to add these as extraction items. They are rarely reported as separate, identifiable quantities, and where they are, they rest on incompatible amortisation periods and definitions of what counts as capital rather than recurrent cost, so the values could not be compared across studies. The review performs no quantitative synthesis — it maps how evaluations are designed and reported, not what they conclude, and does not pool or convert absolute costs — so disaggregated capital and staffing figures would fill fields that no planned analysis would use. What the extraction does capture is the costing approach of each study (micro-costing, TDABC, DRG-based gross costing, tariffs, reimbursement schedules or claims-based data) and whether individual cost components, unit costs and the cost year are reported. We accept that this leaves the capital-intensive character of modern radiotherapy less fully characterised than the reviewer

proposes, and we record it as a deliberate limitation of the extraction framework rather than an oversight.

Comment 8

Separate reporting quality, methodological limitations, and uncertainty.

We agree that these are distinct constructs and that a composite score across them would be uninterpretable. The Quality Appraisal section has been rewritten accordingly (p. 16, line 321 to p. 17, line 331):

> *"Reporting completeness, methodological limitations, and the characterization of uncertainty will be assessed as three separate dimensions and will not be combined into a single overall quality score. Reporting completeness will be assessed using the CHEERS 2022 checklist [53], applied to full economic evaluations, as CHEERS is designed for studies that compare both costs and outcomes. Risk of bias and methodological limitations will be evaluated using the ECOBIAS checklist, applied to decision-analytic model-based economic evaluations [54]. Uncertainty will be assessed using the TRUST framework, applied to the same subset. For partial economic evaluations and budget impact analyses, CHEERS will not be computed, and the number and proportion of studies in each design category will be reported descriptively. The denominator of each appraisal will be reported alongside its results."*

The scope of each instrument therefore follows from the study designs it was built for, and the denominator of each appraisal is reported so that coverage is transparent. Because the three instruments differ in form, they are also scored on their own terms: CHEERS and ECOBIAS use the fully met / partially met / not met scale shown as a traffic light, whereas TRUST is dichotomous by design and records each source of uncertainty as present or absent (p. 17, lines 333–336). Results are presented as three separate item-by-item summaries. The corresponding bullet in 'Strengths and limitations' and the Methods paragraph of the abstract have been revised to match.

Comment 9

Provide a reproducible network-analysis plan. The proposed VOSviewer analysis is insufficiently described.

The previous description named the software but not the analysis. The following has been added to the Synthesis and Evidence Mapping section (p. 18, line 376 to p. 19, line 388):

> *"The co-occurrence matrix will be constructed at the study level from these coded attributes, and the network built and analyzed in R using the igraph package. Nodes represent coded concepts, while edges indicate the co-occurrence of two concepts within the same study, weighted by co-occurrence frequency (association strength). Edges therefore denote joint occurrence rather than economic comparisons, which preserves the publication-level unit of analysis. Only concepts occurring in at least five studies will be retained. Thematic clusters will be identified with a modularity-based community-detection algorithm (the Leiden algorithm [55]), with the random seed fixed in advance and the resolution parameter reported alongside the results. The pairings of primary interest are: radiotherapy modality by type of economic evaluation; and tumour site by radiotherapy modality. The distribution of modalities over publication year will instead be presented as a heatmap, since calendar year is a temporal axis*

rather than a conceptual node. Network maps will be visualized in VOSviewer [56] from the same matrix. The matrix, the input files, and the analysis code will be deposited on OSF."

VOSviewer is kept for visualisation only, and depositing the matrix, input files and code lets the analysis be re-run independently.

Comment 10

Moderate the policy and reimbursement claims. The manuscript states that the review will determine where the evidence is robust enough to support policy and reimbursement decisions. The proposed descriptive methods cannot necessarily support the conclusion.

We agree that the original wording claimed more than a descriptive map can deliver, since establishing that evidence is robust enough for a reimbursement decision would require appraising the validity of the individual estimates. In the Discussion, the passage

> *"the synthesis will enable us to determine where the evidence base is robust enough to support policy and reimbursement decisions and where further methodological work, primary research, or standardization is required. In doing so, evidence mapping will serve not merely as a descriptive overview but also as a strategic tool to inform future research priorities, methodological harmonization efforts, and the design of more rigorous and decision-relevant economic evaluations in radiotherapy."*

has been replaced by (p. 20, line 423 to p. 21, line 428):

> *"the synthesis will show where economic evidence is concentrated and where it is sparse or absent, and where further methodological work, primary research, or standardization is required. In doing so, evidence mapping will inform future research priorities, methodological harmonization efforts, and the design of more rigorous economic evaluations in radiotherapy. Characterizing where the evidence base is robust enough to inform policy or reimbursement decisions, however, falls outside the scope of a descriptive mapping review."*

The words "and decision-relevant" have been removed from the closing clause, and the Methods paragraph of the abstract has been shortened accordingly.

Reviewer's concluding comment

In conclusion, this is a relevant and potentially useful protocol, and the systematic mapping approach is broadly suitable for the research objectives. However, substantial methodological clarification is required to ensure that the review is reproducible and that its eventual conclusions remain proportionate to the evidence generated.

We thank the reviewer for this assessment and for comments that were consistently specific and actionable. The revisions have made the protocol more reproducible and have brought its stated objectives into proportion with what a descriptive map can establish.

VERSION 2 - REVIEW

**Reviewer**	**1**
**Name**	**Ribeiro, Luis**
**Affiliation**	**Universidade do Algarve Escola Superior de Saude, Radiology**
**Date**	**08-Aug-2026**
**COI**	**None**

Dear Authors,

The revised protocol is substantially improved and most of the substantive comments have been addressed. The economic-evaluation taxonomy, reproducible database strategies, publication-level unit of analysis, separation of reporting quality, methodological limitations and uncertainty, and the network-analysis plan are now much clearer. I recommend minor revision before acceptance, limited to the points below.

1. Harmonise the definition of adaptive radiotherapy throughout. The eligibility text includes Magnetic Resonance- or CT-guided adaptive radiotherapy, but Research Question 1 and the Context row of Table 1 still refer only to MR-guided adaptive radiotherapy, while Supplementary File 3 uses the broader but ambiguous category "MR-guided or adaptive RT". Please use one explicit definition in the main text, Table 1 and the extraction form.

2. Correct all supplementary-file numbering and cross-references. The response identifies Supplementary File 1 as the complete search strategies and Supplementary File 2 as the PRISMA-P checklist. In the clean manuscript, however, the Methods section cites the PRISMA-P checklist as Supplementary File 1 and the search strategies as Supplementary File 2, whereas the search-strategy document itself is headed "Supplementary File 1". Please align the manuscript, checklist, file headings and response letter.

3. Update the review stage at resubmission and document the effect of the amendments. The manuscript reports the stage reached on 2 March 2026, but not the stage at the revised submission. Please state whether full-text screening, extraction or appraisal has now begun and confirm that the amended eligibility and classification criteria of 10 July 2026 were applied retrospectively to all records already screened.

4. Add a planned search update, or justify its omission. The searches ended on 24 February 2026, whereas completion is planned for 30 October 2026. A final update of all database searches shortly before synthesis and publication would reduce the risk of omitting recent evaluations; the update date and additional records should be reported.

5. Align the extraction form with the Data Items section. The main text states that reporting of individual cost components, unit costs and cost year will be recorded, but Supplementary File 3 has no explicit unit-cost field. Add this field or revise the text. Separate incremental-cost and incremental-outcome fields would also improve transparency, even if no currency conversion or quantitative pooling is planned.

6. Clarify two methodological wordings. First, avoid the categorical statement that grey literature and HTA sources "cannot be reproduced"; describe their exclusion as a deliberate restriction to the peer-reviewed evidence base and qualify the review aims accordingly. Second, in the network plan distinguish raw co-occurrence frequency from association-strength normalisation, rather than presenting them as equivalent.

Table 1 and Table 2 are legible and methodologically useful, and the supplementary search tables materially improve reproducibility. No empirical figures or graphs are expected at protocol stage; the planned heat maps, bubble plots and conceptual network maps are described adequately, subject to the clarification above.

Kind regards,

VERSION 2 - AUTHOR RESPONSE

REVIEWER 1: Dr Luís Ribeiro

We thank the reviewer for the continued careful examination of the protocol and its supplementary materials. These comments helped us resolve the remaining inconsistencies and improve the clarity and reproducibility of the planned review.

Comment 1

Harmonise the definition of adaptive radiotherapy throughout. The eligibility text includes Magnetic Resonance- or CT-guided adaptive radiotherapy, but Research Question 1 and the Context row of Table 1 still refer only to MR-guided adaptive radiotherapy, while Supplementary File 3 uses the broader but ambiguous category "MR-guided or adaptive RT". Please use one explicit definition in the main text, Table 1 and the extraction form.

We thank the reviewer for identifying this inconsistency. The definition has been harmonised throughout the manuscript and supplementary materials. The eligibility criteria include Magnetic Resonance- or CT-guided adaptive radiotherapy (p. 6, lines 146–148). Research Question 1 now refers to MR- or CT-guided adaptive radiotherapy (p. 7, lines 169–170), and the Context row of Table 1 uses the same wording. The Table 1 footnote defines adaptive radiotherapy as modification of the treatment plan during the treatment course based on on-board MR or CT imaging. Supplementary File 3 now contains separate response options for adaptive RT (MR-guided) and adaptive RT (CT-guided).

Comment 2

Correct all supplementary-file numbering and cross-references. The response identifies Supplementary File 1 as the complete search strategies and Supplementary File 2 as the PRISMA-P checklist. In the clean manuscript, however, the Methods section cites the PRISMA-P checklist as Supplementary File 1 and the search strategies as Supplementary File 2, whereas the search-strategy document itself is headed "Supplementary File 1". Please align the manuscript, checklist, file headings and response letter.

We thank the reviewer for carefully checking the supplementary-file numbering. The discrepancy originated in our previous response letter rather than in the manuscript, and we apologise for it. The numbering and cross-references are now consistent throughout, including in the present letter: Supplementary File 1 is the completed PRISMA-P 2015 checklist, Supplementary File 2 contains the complete search strategies for all five databases, and Supplementary File 3 is the data extraction form. The manuscript cites them at p. 9, lines 186–187, at p. 11, line 222 to p. 12, line 224, and at p. 14, lines 289–291 respectively. The headings of the supplementary files and the internal cross-reference in the PRISMA-P checklist have also been aligned with this numbering.

Comment 3

> *Update the review stage at resubmission and document the effect of the amendments. The manuscript reports the stage reached on 2 March 2026, but not the stage at the revised submission. Please state whether full-text screening, extraction or appraisal has now begun and confirm that the amended eligibility and classification criteria of 10 July 2026 were applied retrospectively to all records already screened.*

We thank the reviewer for requesting a clearer account of the review stage. The Study Selection section now reports the stage reached as of 31 August 2026 (p. 12, lines 237–238): the first reviewer has screened all 6,883 records, while the second reviewer is still working on the screening. The Abstract states that study selection is ongoing, that extraction and quality appraisal in duplicate have not been undertaken, and that no results are reported (p. 2, lines 44–45).

The manuscript further confirms that all records screened before 10 July 2026 were re-examined after the eligibility definition was extended to CT-guided adaptive radiotherapy (p. 12, lines 238–240). The amended taxonomy of economic evaluation designs operates at a different stage: because classification is performed at extraction, no study has been classified under the superseded taxonomy, and the amended taxonomy will govern the classification of every included study, so that no retrospective reclassification is required (p. 12, lines 240–241).

Comment 4

> *Add a planned search update, or justify its omission. The searches ended on 24 February 2026, whereas completion is planned for 30 October 2026. A final update of all database searches shortly before synthesis and publication would reduce the risk of omitting recent evaluations; the update date and additional records should be reported.*

We thank the reviewer for this important suggestion. We have added a planned update of all five database searches before synthesis, using the same strategies and the same interface limiters as the original searches, restricted to records indexed after 24 February 2026 and deduplicated against the existing set. The date of the updated search, the number of additional records retrieved after deduplication, and the number of additional studies included will be reported in the

PRISMA flow diagram and in the completed review (p. 12, lines 224–228). The eligibility period now refers to the final search date of 24 February 2026 together with the planned update before synthesis (p. 7, lines 157–159). This change will be documented as a dated amendment in the OSF registry.

Comment 5

> *Align the extraction form with the Data Items section. The main text states that reporting of individual cost components, unit costs and cost year will be recorded, but Supplementary File 3 has no explicit unit-cost field. Add this field or revise the text. Separate incremental-cost and incremental-outcome fields would also improve transparency, even if no currency conversion or quantitative pooling is planned.*

We thank the reviewer for identifying this inconsistency. Supplementary File 3 now includes an explicit "Unit costs reported" item ("Yes (unit costs and quantities separable) / Partially / No / Not reported"), aligned with the Data Items section (p. 13, line 273 to p. 14, line 274).

We have also adopted the second suggestion, in the form our mapping objectives can support: Supplementary File 3 now records whether incremental costs and incremental outcomes are reported ("Both / Costs only / Outcomes only / Neither"), alongside the existing items on reported cost values, outcomes, ICER presence and ICER value in its original units. We have recorded their presence rather than their values because an incremental estimate is a property of the result rather than of the design, whereas this review maps how evaluations are designed and reported (p. 16, lines 324–329). Whether such estimates are reported is itself a reporting characteristic, and complements the CHEERS-based assessment.

Comment 6, first part

> *Avoid the categorical statement that grey literature and HTA sources "cannot be reproduced"; describe their exclusion as a deliberate restriction to the peer-reviewed evidence base and qualify the review aims accordingly.*

We thank the reviewer for this correction. We agree that the previous statement was too categorical and have removed it. The Methods section now states (p. 9, lines 195–198):

> "This review is deliberately restricted to the peer-reviewed evidence base; grey literature and health technology assessment (HTA) reports were not searched. This restriction provides a clearly defined corpus but may omit economic methods used in commissioned or jurisdiction-specific decision-making contexts."

The Abstract and Introduction now explicitly define the review as a characterisation of the published, peer-reviewed evidence base (p. 2, lines 33–36; p. 6, line 125). The Discussion retains the exclusion of HTA reports and other grey literature as a limitation (p. 19, lines 403–405).

Comment 6, second part

> *In the network plan distinguish raw co-occurrence frequency from association-strength normalisation, rather than presenting them as equivalent.*

We thank the reviewer for identifying the conflation of these measures. The Synthesis and Evidence Mapping section now distinguishes raw co-occurrence frequency, defined as the

number of publications in which two concepts occur together, from association strength, which normalises the observed co-occurrence by the marginal frequencies of the two concepts (p. 17, lines 349–354). The raw co-occurrence matrix will be deposited on OSF, while community detection and the network layouts will use association-strength weights, and the weighting will be stated in each figure legend. VOSviewer visualisations will use the same association-strength normalisation, so that both the R and VOSviewer representations are based on the same normalised edge weights (p. 17, lines 361–363).

We thank Dr Luís Ribeiro for the detailed and constructive comments and the Editor for the opportunity to revise the manuscript.
