# Supplementary material for: Methods and evidence in economic evaluations of modern radiotherapy: a systematic mapping review protocol
Source: BMJ Open. 2026 Sep 22;16(9):e119043. doi: 10.1136/bmjopen-2026-119043 (PMC13599855; doi:10.1136/bmjopen-2026-119043)
Supplement: Supplementary file 2 [file bmjopen-16-9-s001.docx]

**PRISMA-P (Preferred Reporting Items for Systematic review and Meta-Analysis Protocols) 2015 checklist: recommended items to address in a systematic review protocol***

**Supplementary File 1**

Manuscript bmjopen-2026-119043. Page and line numbers refer to the clean revised manuscript submitted with this revision.

| **Section and topic** | **Item No** | **Checklist item** | **Reported on page / line No.** |
| --- | --- | --- | --- |
| **ADMINISTRATIVE INFORMATION** |  |  |  |
| **Title:** |  |  |  |
| Identification | 1a | Identify the report as a protocol of a systematic review | p. 1, lines 2–3. The title identifies the report as a systematic mapping review protocol. |
| Update | 1b | If the protocol is for an update of a previous systematic review, identify as such | Not applicable. This protocol is not an update of a previously completed or published review. |
| Registration | 2 | If registered, provide the name of the registry (such as PROSPERO) and registration number | p. 3, lines 61–62 (Abstract – Registration) and p. 19, line 421 to p. 20, line 427 (Amendments). Open Science Framework Registries, DOI 10.17605/OSF.IO/6W2TJ, registered prospectively on 9 February 2026. |
| **Authors:** |  |  |  |
| Contact | 3a | Provide name, institutional affiliation, e-mail address of all protocol authors; provide physical mailing address of corresponding author | p. 1, lines 5–20 (author names and affiliations); p. 1, line 22 to p. 2, line 25 (corresponding author and postal address); p. 2, line 26 (e-mail address). |
| Contributions | 3b | Describe contributions of protocol authors and identify the guarantor of the review | p. 20, lines 439–444 (Author Contributions). FV is identified as guarantor at lines 443–444. |
| Amendments | 4 | If the protocol represents an amendment of a previously completed or published protocol, identify as such and list changes; otherwise, state plan for documenting important protocol amendments | p. 19, line 421 to p. 20, line 427 (Amendments). Two amendments (11 February 2026 and 10 July 2026) are listed, with a stated plan for documenting any further amendments on OSF. |
| **Support:** |  |  |  |
| Sources | 5a | Indicate sources of financial or other support for the review | p. 20, lines 446–448 (Funding). European Union, Marie Skłodowska-Curie Actions Doctoral Network UPLIFT. |
| Sponsor | 5b | Provide name for the review funder and/or sponsor | p. 20, line 448 (Funding). Grant Agreement No. 101168955. |
| Role of sponsor or funder | 5c | Describe roles of funder(s), sponsor(s), and/or institution(s), if any, in developing the protocol | p. 20, line 448 to p. 21, line 451 (Funding). The funder had no role in the design, conduct or reporting of the review. |
| **INTRODUCTION** |  |  |  |
| Rationale | 6 | Describe the rationale for the review in the context of what is already known | p. 4, line 85 to p. 6, line 134 (Introduction), including the gap left by earlier single-modality, single-site and narrowly methodological reviews at p. 5, lines 117–124. |
| Objectives | 7 | Provide an explicit statement of the question(s) the review will address with reference to participants, interventions, comparators, and outcomes (PICO) | p. 2, lines 35–39 (Abstract – aims); p. 6, lines 129–134 (objectives i–iii); p. 7, line 167 to p. 8, line 179 (research questions). Eligibility is framed using the PCC framework in Table 1 rather than PICO, as is conventional for mapping reviews. |
| **METHODS** |  |  |  |
| Eligibility criteria | 8 | Specify the study characteristics (such as PICO, study design, setting, time frame) and report characteristics (such as years considered, language, publication status) to be used as criteria for eligibility for the review | p. 6, line 137 to p. 8, line 179 (Eligibility criteria and research questions), with Table 1 on p. 8. Study-design taxonomy at p. 6, lines 137–143; definition of modern radiotherapy and eligible comparators at p. 6, lines 146–148; period, language and publication status at p. 7, lines 157–160. |
| Information sources | 9 | Describe all intended information sources (such as electronic databases, contact with study authors, trial registers or other grey literature sources) with planned dates of coverage | p. 9, lines 193–198 (Databases and Search Strategy): five databases, coverage from 1 January 2005 to 24 February 2026, and the deliberate restriction to the peer-reviewed evidence base. Citation tracking and author- and journal-based searching at p. 11, lines 218–222; planned update of all database searches before synthesis at p. 12, lines 224–228. |
| Search strategy | 10 | Present draft of search strategy to be used for at least one electronic database, including planned limits, such that it could be repeated | p. 10, line 212 and Table 2 (pp. 10–11): worked PubMed strategy with fields, MeSH terms and limits. The verbatim strategies as run for all five databases are provided in Supplementary File 2, referenced at p. 11, line 222 to p. 12, line 224. |
| **Study records:** |  |  |  |
| Data management | 11a | Describe the mechanism(s) that will be used to manage records and data throughout the review | p. 12, lines 231–233 (Study Selection). Zotero for reference management, ASySD for deduplication and Rayyan for screening. |
| Selection process | 11b | State the process that will be used for selecting studies (such as two independent reviewers) through each phase of the review (that is, screening, eligibility and inclusion in meta-analysis) | p. 12, lines 244–248 (Study Selection). Two reviewers screen independently at both stages, disagreements are resolved by discussion or a third reviewer, and inter-rater agreement is reported using Cohen’s kappa for the pilot phase (lines 242–243). The stage reached at this revision and the retrospective application of the amended eligibility criteria are reported at p. 12, lines 236–241. |
| Data collection process | 11c | Describe planned method of extracting data from reports (such as piloting forms, done independently, in duplicate), any processes for obtaining and confirming data from investigators | p. 13, lines 257–259 (Data Items). A standardised extraction form is pilot-tested and data are extracted independently in duplicate by two reviewers. |
| Data items | 12 | List and define all variables for which data will be sought (such as PICO items, funding sources), any pre-planned data assumptions and simplifications | p. 13, lines 259–261 (four descriptive domains) and p. 13, line 262 to p. 14, line 291 (Data Items). The draft extraction form is given in Supplementary File 3, referenced at p. 14, lines 289–291. |
| Outcomes and prioritization | 13 | List and define all outcomes for which data will be sought, including prioritization of main and additional outcomes, with rationale | p. 7, line 167 to p. 8, line 179 (research questions) and p. 13, line 257 to p. 14, line 291 (Data Items). Adapted for a mapping review: the mapped dimensions serve as the review outcomes. The primary focus is the distribution and methodological characterisation of the evidence (RQ1 and RQ2); the handling of uncertainty, including the TRUST classification, is secondary. |
| Risk of bias in individual studies | 14 | Describe anticipated methods for assessing risk of bias of individual studies, including whether this will be done at the outcome or study level, or both; state how this information will be used in data synthesis | p. 14, line 293 to p. 15, line 319 (Quality Appraisal). CHEERS 2022 is applied to full economic evaluations, ECOBIAS and TRUST to decision-analytic model-based evaluations. Appraisal is at study level, reported descriptively as three separate dimensions, and is not used as an inclusion criterion. The denominator of each appraisal is reported (p. 15, lines 305–306). |
| Data synthesis | 15a | Describe criteria under which study data will be quantitatively synthesised | p. 15, lines 322–323 (Synthesis and Evidence Mapping). Quantitative synthesis is not undertaken; heterogeneity in study design, clinical context and evaluation framework precludes meta-analysis. |
|  | 15b | If data are appropriate for quantitative synthesis, describe planned summary measures, methods of handling data and methods of combining data from studies, including any planned exploration of consistency (such as I², Kendall’s τ) | Not applicable. No quantitative synthesis or meta-analysis is planned. |
|  | 15c | Describe any proposed additional analyses (such as sensitivity or subgroup analyses, meta-regression) | p. 16, lines 329–334 (evidence gap maps, heatmaps, bubble plots and matrix-based visualisations) and p. 16, line 335 to p. 17, line 363 (conceptual co-occurrence network built at study level in R with igraph, with raw co-occurrence frequency and association strength reported separately, Leiden community detection with a fixed seed, visualised in VOSviewer; matrix, input files and code deposited on OSF). |
|  | 15d | If quantitative synthesis is not appropriate, describe the type of summary planned | p. 15, line 322 to p. 16, line 334 (Synthesis and Evidence Mapping). Descriptive tabulation and narrative synthesis, supplemented by evidence gap maps, heatmaps, bubble plots and matrix-based visualisations. |
| Meta-bias(es) | 16 | Specify any planned assessment of meta-bias(es) (such as publication bias across studies, selective reporting within studies) | p. 15, lines 317–318 (Quality Appraisal). No formal statistical assessment of publication bias across studies will be undertaken, and the reason is stated. |
| Confidence in cumulative evidence | 17 | Describe how the strength of the body of evidence will be assessed (such as GRADE) | p. 15, lines 318–319 (Quality Appraisal). No formal assessment of confidence in cumulative evidence, such as GRADE, will be performed, and the reason is stated. |

*** It is strongly recommended that this checklist be read in conjunction with the PRISMA-P Explanation and Elaboration for important clarification on the items. Amendments to a review protocol should be tracked and dated. The copyright for PRISMA-P (including checklist) is held by the PRISMA-P Group and is distributed under a Creative Commons Attribution Licence 4.0.**

*From: Shamseer L, Moher D, Clarke M, Ghersi D, Liberati A, Petticrew M, Shekelle P, Stewart L, PRISMA-P Group. Preferred reporting items for systematic review and meta-analysis protocols (PRISMA-P) 2015: elaboration and explanation. BMJ. 2015 Jan 2;349(jan02 1):g7647.*
