# Supplementary material for: Methods and evidence in economic evaluations of modern radiotherapy: a systematic mapping review protocol
Source: BMJ Open. 2026 Sep 22;16(9):e119043. doi: 10.1136/bmjopen-2026-119043 (PMC13599855; doi:10.1136/bmjopen-2026-119043)
Supplement: Supplementary file 3 [file bmjopen-16-9-s003.docx]

**Supplementary File 3**

*Draft data extraction items*

Methods and Evidence in Economic Evaluations of Modern Radiotherapy — Veneruso et al. (BMJ Open, ID bmjopen-2026-119043). Data extraction form (pilot version).

| **Variable** | **Response categories / description** |
| --- | --- |
| **Study characteristics** | |
| **Author, year** | First author and year of publication |
| **Country** | Country where the study was conducted |
| **Geographical region** | World Bank regional grouping |
| **Income group** | World Bank income group (High / Upper-middle / Lower-middle / Low) |
| **Funding source** | Industry / Public / Mixed / Not reported |
| **Conflict of interest** | Yes / No / Not reported |
| **Study design** | Trial-based / Model-based / Observational |
| **Clinical context** | |
| **Tumour site** | Harmonized category and site as reported |
| **Disease stage** | Stage as reported |
| **Treatment intent** | Curative / Palliative / Mixed / Not reported |
| **Concomitant systemic therapy** | RT alone / Concurrent / Induction / Adjuvant / Not reported |
| **Population** | Narrative description of patients or modelled cohort, including age, sex and ethnicity where reported |
| **Sample size / modelled cohort** | Number of patients or simulated cohort size, with observed vs modelled cohort noted |
| **Care setting** | Hospital / Outpatient / National programme / Multicentre / Other |
| **Radiotherapy characteristics** | |
| **Modality evaluated** | IMRT / VMAT / SBRT / SRS / Proton / Carbon ion / adaptive RT (MR-guided) / adaptive RT (CT-guided); combinations recorded as mixed-modality |
| **Comparator** | 3D-CRT / alternative modern RT modality / non-RT comparator / no treatment or watchful waiting / multiple comparators / not reported |
| **Fractionation details** | Dose, fractions, planning characteristics (where reported) |
| **Economic evaluation** | |
| **Type of evaluation** | Full EE: CUA / CEA / CBA / CMA / cost-consequence. Partial EE: cost analysis (comparative or single-arm). Budget impact analysis (classified separately). Multiple (more than one design in one study) |
| **Analytical perspective** | Healthcare payer / Hospital / Societal / Other |
| **Time horizon** | Standardized band and as reported (treatment period only / short-term <2y / medium-term 2-9y / long-term ≥10y / lifetime / NA / NR) |
| **Discount rate (costs)** | Rate applied to costs (%), or not reported / not applicable |
| **Discount rate (outcomes)** | Rate applied to outcomes (%), or not reported / not applicable |
| **Model structure** | Not applicable (trial-based) / decision tree / Markov / microsimulation / hybrid or other |
| **Costing approach** | |
| **Costing method** | Micro-costing / TDABC / DRG-based gross costing / tariffs / reimbursement schedules / claims-based / mixed |
| **Cost components reported** | Yes / Partially / No |
| **Unit costs reported** | Yes (unit costs and quantities separable) / Partially / No / Not reported |
| **Cost year reported** | Yes / No |
| **Currency** | Original currency, as reported |
| **Cost year** | As reported |
| **Reported cost values** | Extracted in original currency and cost year, without standardisation (e.g., total cost, cost per fraction, cost per patient or treatment course) |
| **Outcomes** | |
| **Outcomes reported** | Costs / QALYs / LYG / DALYs / ICER / Other |
| **ICER reported** | Yes / No |
| **ICER value** | As reported (original currency and outcome unit) |
| **Incremental costs and outcomes reported** | Both / Costs only / Outcomes only / Neither |
| **Effectiveness source** | Randomized trial / observational study / meta-analysis / expert opinion / assumption |
| **WTP or cost-effectiveness threshold** | Presence and type where reported (fixed jurisdictional value / GDP-based / none reported) |
| **Utility measurement** | |
| **Utility method** | EQ-5D-3L or 5L / SF-6D / TTO / SG / disease-specific measure / mapping from non-preference instrument / literature values / not applicable / not reported |
| **Utility value set / tariff and jurisdiction** | Population value set or tariff applied and its jurisdiction, where reported |
| **Uncertainty** | |
| **Uncertainty methods** | None / deterministic sensitivity analysis / probabilistic sensitivity analysis / scenario / threshold / value of information |
| **TRUST classification** | Parameter / structural / methodological (decision-analytic model-based studies only); not applicable otherwise |
